# Selection, characterization, and thermal stabilization of llama single domain antibodies towards Ebola virus glycoprotein

**DOI:** 10.1186/s12934-017-0837-z

**Published:** 2017-12-12

**Authors:** Jinny L. Liu, Lisa C. Shriver-Lake, George P. Anderson, Dan Zabetakis, Ellen R. Goldman

**Affiliations:** US Naval Research Laboratory, Center for Bio/Molecular Science and Engineering, 4555 Overlook Ave SW, Washington, DC 20375 USA

**Keywords:** Single domain antibodies, Antibody engineering, Ebola virus, Glycoprotein, Virus like particles

## Abstract

**Background:**

A key advantage of recombinant antibody technology is the ability to optimize and tailor reagents. Single domain antibodies (sdAbs), the recombinantly produced variable domains derived from camelid and shark heavy chain antibodies, provide advantages of stability and solubility and can be further engineered to enhance their properties. In this study, we generated sdAbs specific for Ebola virus envelope glycoprotein (GP) and increased their stability to expand their utility for use in austere locals. Ebola virus is extremely virulent and causes fatal hemorrhagic fever in ~ 50 percent of the cases. The viral GP binds to host cell receptors to facilitate viral entry and thus plays a critical role in pathogenicity.

**Results:**

An immune phage display library containing more than 10^7^ unique clones was developed from a llama immunized with a combination of killed Ebola virus and recombinantly produced GP. We panned the library to obtain GP binding sdAbs and isolated sdAbs from 5 distinct sequence families. Three GP binders with dissociation constants ranging from ~ 2 to 20 nM, and melting temperatures from ~ 57 to 72 °C were selected for protein engineering in order to increase their stability through a combination of consensus sequence mutagenesis and the addition of a non-canonical disulfide bond. These changes served to increase the melting temperatures of the sdAbs by 15–17 °C. In addition, fusion of a short positively charged tail to the C-terminus which provided ideal sites for the chemical modification of these sdAbs resulted in improved limits of detection of GP and Ebola virus like particles while serving as tracer antibodies.

**Conclusions:**

SdAbs specific for Ebola GP were selected and their stability and functionality were improved utilizing protein engineering. Thermal stability of antibody reagents may be of particular importance when operating in austere locations that lack reliable refrigeration. Future efforts can evaluate the potential of these isolated sdAbs as candidates for diagnostic or therapeutic applications for Ebola.

**Electronic supplementary material:**

The online version of this article (10.1186/s12934-017-0837-z) contains supplementary material, which is available to authorized users.

## Background

Camelid single domain antibodies (sdAbs, also referred to as nanobodies or VHH) and shark-derived sdAbs (termed VNAR) are recombinantly expressed variable domains derived from the heavy-chain-only antibodies found in camelids and sharks [1–5]. Each sdAb contains three variable complementarity determining regions (CDRs) and four relatively well conserved framework regions (FRs). SdAbs constitute the smallest naturally occurring antigen binding domains and offer many advantages. Their superior thermal stability and ability to recognize hidden epitopes combined with specificity and binding affinity comparable to conventional antibodies (Abs) make sdAbs an attractive alternative to Abs [6–9]. Importantly, sdAbs are amenable to protein engineering to improve their properties. While sdAbs are generally thermal stable and most refold and recover the majority of their secondary structure after heat denaturation, sdAbs are not heat proof. They can, however, be engineered to increase their melting temperature [10]. Furthermore, sdAbs can be engineered to tailor them for integration into specific applications [6, 11]. These properties make sdAbs ideal reagents for a wide range of uses including detection of, or therapeutic response to, infectious diseases and bio-threat agents [6–9, 11, 12].

An important target for sdAb development is the Ebola virus. Ebola virus is a member of Family Filoviridae, along with the Marburg viruses. Genus Ebola virus includes four species, *Zaire ebolavirus* (EBOV)*, Sudan ebolavirus*, (SUDV) *Tai Forest ebolavirus* (TAFV) and *Bundibugyo ebolavirus* (BDBV) originally described in Africa plus *Reston ebolavirus* (RESTV) from the Philippines. With the exception of Reston virus, these viruses cause severe fatal viral hemorrhagic fever due to the systemic infection and replication upon entry into the human body and if untreated result in mortality rates of up to 90%. The incubation period for EBOV infection lasts 5–7 days and in most of patients symptoms appear within 21 days after the exposure. In some cases the infection of Ebola viruses can be detected as early as 2 days post exposure by reverse transcription polymerase chain reaction (RT-PCR) [13]. In addition, enzyme-linked immunosorbent assays (ELISAs) are used for the detection of Ebola specific IgM and IgG antibodies and viral antigens. Viral particles detected by antibodies can be traced in blood from day 3 up to day 7–16 following the beginning of symptoms. While there are a number of promising leads, currently there is no licensed vaccine nor an approved treatment available for human use (http://www.who.int/mediacentre/factsheets/fs103/en/); [14].

The cause of the extreme virulence by Ebola virus infection is still not well established. However current data suggests that the interaction of glycosylated envelope protein (glycoprotein) with the immune system plays an important role in the extraordinary pathogenicity of this virus. The Ebola virus genome consists of a non-segmented, negative sense RNA, approximately 19 kb in size, which encodes seven structural proteins and two glycoproteins (GPs), the envelope glycoprotein (GP) and a secretory GP (sGP) [15, 16]. Transcriptional editing of the *GP* gene results in production of a transmembrane-linked GP with a length of 676 amino acids and a secreted soluble non-structural sGP with a length of 364 amino acids [17]. Both GP and sGP share approximately 300 N-terminal amino acids; sGP is detected in high concentrations in the blood of acutely infected patients. The role of sGP is not well known yet, but likely it is involved in limiting neutrophil activation by binding to host receptors and inhibiting the neutralizing activity of anti-GP antibodies by decoying them. Most Ebola virus GP contains a convertase furin cleavage site, which results in two subunits, GP1 and GP2 in the native GP [18]. GP1 and GP2 form heterodimers on the virions. The surface subunit (GP1) is involved in receptor binding and the transmembrane subunit (GP2) mediates the virus host membrane fusion. Ebola virus GP is responsible for the entry of viruses to the target cells and thus is considered the most important protein for pathogenesis. It is the sole protein on the viral surface and serves as the primary target of neutralizing antibodies.

Several promising therapeutic antibodies specific against GP, including humanized mouse antibodies (Zmapp), bi-specific antibodies and other related monoclonal antibodies aiming to neutralize Ebola viruses by inhibiting viral entry to host cells have been or are under development [19–21]. SdAbs represent attractive alternatives to these conventional antibodies.

Both llama- and shark-derived sdAbs specific for the EBOV nucleoprotein (NP) have been described for the detection of EBOV [22, 23]. However, sdAbs against EBOV GP have not yet been developed. In this study, a combination of killed EBOV and recombinant EBOV GP was used to immunize a llama and an immune sdAb library was developed for biopanning against EBOV GP. Specific GP binders were selected and characterized for binding to GP and EBOV virus like particles (VLPs). The sequences of the EBOV GP binding sdAbs were modified to improve their physical properties and the detection capability for GP and VLPs.

## Results and discussion

### Library construction and biopanning

Ideally an immune sdAb phage display library captures the repertoire of variable regions from the heavy-chain-only antibodies of an immunized animal. A library of phage displayed sdAbs derived from a llama immunized with both killed EBOV and recombinant EBOV GP was constructed. As described in the methods, the variable domains amplified from heavy-chain-only antibodies were cloned into the phage display vector pECAN 21 and the ligated vectors were subsequently transformed into *E. coli* through electroporation. Twenty colonies were randomly picked from the plates and sequenced. Approximately 90%, 18 out of 20 clones, had unique and full length sequences. In addition, the library was subject to deep sequencing and ~ 1,180,000 sequences were recovered. A random sample of 100 sequences were checked for copy number and of these, 93% were present in the library as a single copy. Library size was estimated using the number of transformants in combination with the percentage of unique sequences. From this calculation it was estimated that the constructed EBOV GP immune phage display library contained 1.8 × 10^7^ unique representatives, a large enough collection of variable domains for subsequent rounds of biopanning and selection.

For each round of biopanning, we used approximately 5 × 10^11^ phage to ensure multiple copies of each sdAb sequence were included. Three rounds of panning were performed and resulted in 10- and 24-fold enrichment in phage titers relative to the respective previous rounds, round 2 (R2) and round 3 (R3), as indicated in Additional file 1: Figure S1. Approximately 198 colonies from R2 and R3 were subjected to monoclonal phage ELISA (data not shown). Forty clones with absorbance ratios (EBOV GP/BSA) between 4 and 80 were sequenced; the thirty-four unique sequences were grouped into sequence families based on CDR homology and are shown in Additional file 1: Figure S2.

**Fig. 1:**
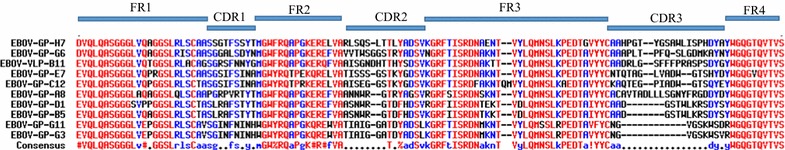
Representative EBOV GP binding sdAbs selected after three rounds of biopannings. The sdAbs were divided into sequence families based on similarity of their CDRs. These 10 sdAbs include representatives from the different families. Each of these sdAb clones was produced and characterized

We chose 10 representative clones that spanned the identified sequence families to subclone into pET22b for protein expression (Fig. 1). The expressed protein was purified by immobilized metal affinity chromatography (IMAC) and gel filtration. The yields of sdAbs ranged from 2 to 20 mg/L of cell culture, sufficient for subsequent measurements.

### Characterization of initially selected sdAb

After preparing protein, the melting temperatures (Tm) of 10 sdAbs representing several distinct sequence families were measured using a fluorescent dye melt assay (Table 1). Tms ranged from 46 to 68 °C, with the highest values measured for EBOV-GP-H7 and EBOV-GP-G6.

**Table 1:** Binding affinity and melting temperature (Tm) for EBOV GP binding sdAb

Clone name	Tm (°C)	ka (1/Ms)	kd (1/s)	K_D_ (M)
EBOV-GP-E7	54	3.5E+03	1.1E−04	3.4E−08
EBOV-GP-G6	67	7.0E+04	1.5E−04	2.0E−09
EBOV-GP-H7	68	1.7E+04	3.0E−04	1.7E−08
EBOV-GP-A8	60	3.1E+04	7.2E−05	2.3E−09
EBOV-GP-D1	51	3.0E+04	3.3E−04	1.1E−08
EBOV-GP-B5	60	3.4E+04	3.7E−04	1.1E−08
EBOV-GP-C12	62	3.28E+03	8.71E−05	2.68E−08
EBOV-GP-G11	46	No binding	No binding	No binding
EBOV-GP-G3	61	No binding	No binding	No binding
EBOV-GP-B11	62	8.98E+04	3.21E−03	3.59E−08

Ten sdAbs representing the sequence families identified in selections for EBOV GP were characterized in terms of melting temperature and binding ability. Melting temperatures were determined by a fluorescent dye melt assay. Binding kinetics were measured by surface plasmon resonance (SPR)

We examined the ability of the 10 selected binders to bind immobilized GP. The on-rate and off-rate (ka and kd respectively) were measured using the ProteOn XPR36, a surface plasmon resonance (SPR) based biosensor (Additional file 1: Figure S3; Table 1) and the dissociation constant (K_D_) determined for each putative GP binder. Measurements were performed in duplicate and standard deviations from the calculated curve fit were obtained. EBOV-GP-A8 has the slowest off rate, which results in one of the lowest K_D_ values among the GP sdAbs, displaying ~ 2 nM affinity. Another GP sdAb, EBOV-GP-G6 also exhibited ~ 2 nM affinity for EBOV GP. Clones EBOV-GP-G3 and EBOV-GP-G11, both belonging to the same sequence family, failed to bind GP in these experiments. The other sdAbs had K_D_ values ranging from 11 to 34 nM. The two clones that showed no binding are part of a sequence family that contained five representatives identified through monoclonal phage ELISA. Differences in antigens attached versus adsorbed to a surface can affect the availability of binding sites. Thus it seems more likely that their epitope on GP is obscured during the covalent immobilization process as opposed to being false positives. Using SPR binding competition for GP, it was observed that EBOV-GP-A8 competed with EBOV-GP-G6, EBOV-GP-H7, EBOV-GP-D1, EBOV-GP-B5, and EBOV-GP-B11; but did not show competition with EBOV-GP-E7 or EBOV-GP-C12 (Additional file 1: Figure S5).

### Sequence modifications of three GP binding sdAbs

The two sdAbs with the best binding characteristics, EBOV-GP-A8 and EBOV-GP-G6 along with the sdAb with the highest Tm, EBOV-GP-H7, were selected for sequence modification to improve their thermal stability. Our approach to thermal stabilization is to utilize a process that combines a number of strategies including addition of a disulfide bond and point mutations, to achieve the end result of a construct with an improved Tm. We use the IMGT numbering scheme [24] to indicate specific positions within each sdAb. The antigen receptor numbering and receptor classification tool [25] was used to number amino acid sequences of the sdAbs and a table of position number and corresponding amino acid for clones EBOV-GP-A8, EBOV-GP-H7 and EBOV-GP-G6 is shown as Additional file 1: Figure S4.

A non-canonical disulfide bond was added to the three sdAbs by incorporating two Cys within FR2 and FR3 at positions 54 and 78 respectively. Previous studies showed that the introduction of a disulfide bond at this location significantly increases Tm, with gains of 4–19 °C and can also increase the proteolytic stability of sdAbs [26–31].

**Fig. 2:**
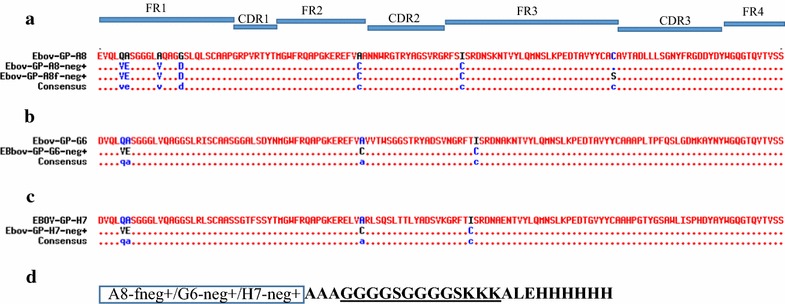
Sequence modification to enhance physicochemical properties. **a** EBOV-GP-A8-neg+ sequence has four amino acid substitutions within FR1 (Q5V, A6E, A13V, and G17D), while EBOV-GP-G6-neg+ in **b** and EBOV-GP-H7-neg+ in **c** have only two substitutions in FR1, Q5V, A6E. All of the mutants have an insertion of a disulfide bond formed by two substituted Cys, A54C and I78C, within FR2 and FR3 indicated in **a**-**c**. EBOV-GP-A8-fneg+ sequence in **a** has the same sequence as EBOV-GP-A8-neg+ except with replaced C106S, which eliminates an unpaired Cys thus disrupting the potential formation of an inter-disulfide bond. **d** The underlined GGGGSGGGGKKK (GSKKK) sequence in bold font is fused onto the C-terminus of EBOV-GP-A8-fneg+, EBOV-GP-G6-neg+, and EBOV-GP-H7-neg+. Following each clone name the suffix “neg” indicates mutations in FR1 that result in the addition of negative charges, and the “+” indicates the addition of one disulfide bond formed by two substituted Cys in FR2 and FR3. The “f” denotes “fixing” the EBOV-GP-A8 sdAb through mutating an unpaired Cys to Ser in FR3 as indicated in **a**

**Fig. 3:**
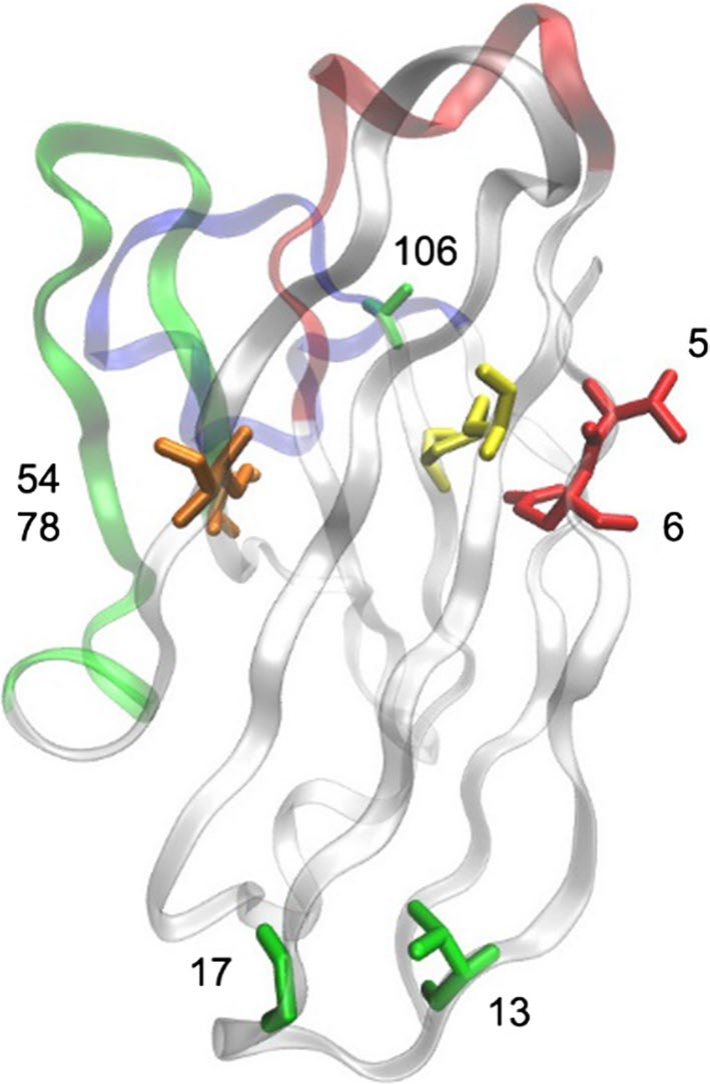
Mutations mapped onto the sdAb structure. The ribbon structure is transparent with the CDRs colored red, green and blue for CDR 1, 2, and 3 respectively. The yellow sticks are the canonical disulfide. The orange sticks are the residues that were changed to make the new disulfide. The red sticks are the VE residues near the N-terminus. The green sticks are the other changes made to EBOV-GP-A8-fneg+. Positions that have been changed by mutagenesis are indicated with the IMGT number of the amino acid that was mutated. The base structure (PDB 5LZ0) is a llama sdAb [50]

In addition to the Cys mutations, negatively/neutrally charged residues were placed within FR1 which resulted in the addition of more negative charges in sdAb mutants relative to the parental sdAbs (Table 2). Clones with the point mutations in FR1 and the added Cys pair are designated by the suffix “-neg+”. Both EBOV-GP-G6-neg+ and EBOV-H7-neg+ have two amino acid changes (Q5V, A6E) in FR1, while EBOV-GP-A8-neg+ has four changes (Q5V, A6E, A13V, G17D) within FR1 (Fig. 2a–c). Table 2 provides a guide to the variants we constructed and characterized. A ribbon structure is presented in Fig. 3 to show where the mutations are located in the three-dimensional sdAb structure.

**Table 2:** Description of sdAbs and variants

Clone name	Description of mutations	Charge Δ
EBOV-GP-G6	Original clone	0
EBOV-GP-G6-neg+	Disulfide addition: A54C, I78C	− 1
	FR1 changes: Q5V, A6E	
EBOV-GP-H7	Original clone	0
EBOV-GP-H7-neg+	Disulfide addition: A54C, I78C	− 1
	FR1 changes: Q5V, A6E	
EBOV-GP-A8	Original clone	0
EBOV-GP-A8-neg+	Disulfide addition: A54C, I78C	− 2
	FR1 changes: Q5V, A6E, A13V, G17D	
EBOV-GP-A8-fneg+	Disulfide addition: A54C, I78C	− 2
	FR1 changes: Q5V, A6E, A13V, G17D	
	Unpaired Cys mutated: C106S	

Three of the GP binding sdAbs showing the highest melting temperatures and/or best affinities were subject to mutagenesis to improve their stability. Mutations included addition of a non-canonical disulfide bond, changes to FR1 that added negative charge, and “fixing” clone EBOV-GP-A8 by mutating an unpaired Cys

The site directed mutagenesis was guided by sequences of sdAbs with high Tms. Previously, we had shown that the introduction of the sequence VE at positions 5 and 6 of an sdAb in combination with 1E or D, and 3Q could lead to an increase in Tm of 5–9 °C [32, 33]. Other researchers examined a large repertoire of sdAb sequences and identified the changes 1E and 5V as stabilizing [34]. Also, prior studies have shown that the addition of negative charges can increase thermal stability and solubility of sdAbs [35, 36].

When examining the sequences of the sdAbs we had noted that EBOV-GP-A8, and its whole sequence family contained an unpaired Cys within FR3. We constructed clone EBOV-GP-A8-fneg+ containing the Cys substitutions at positions 54 and 78 plus the negative changes in FR1 with the additional change, C106S, to eliminate the unpaired non-canonical Cys, which might form an inter-molecular disulfide bridge. Previously, we as well as other researchers have eliminated unpaired Cys within sdAbs with the resulting variants retaining the binding ability of the parental sdAb [37, 38].

We also incorporated a modification of the sdAbs designed to increase their performance in assays. A positively charged tail consisting of a linker and three lysine residues, termed GSKKK (or GS3K), and consisting of the sequence GGGGSGGGGKKK, was genetically fused to the C-terminus of the three sdAbs (Fig. 2d). We hypothesized the tail would serve to increase biotin incorporation or facilitate directional immobilize of the sdAbs.

### Characterization of sdAb variants

Circular dichroism (CD) spectroscopy was performed to measure the Tm and refolding capacity of the modified and parental sdAbs. The native secondary structure of peptides exhibits a maximum ellipticity (mdeg) at lower temperature. As the sdAb is heated, the ellipticity decreases due to loss of the secondary structure accompanied with the unfolding of the tertiary structure as the protein reaches its Tm. The temperature corresponding to the inflection point of the S-shaped curve is defined as the Tm. Refolding percentage is calculated as the percentage of the ellipticity recovered for the cooled protein compared to total change in ellipticity of the protein during the heating cycle. Our results show that the modified sdAbs display increases in Tm of 15–17 °C compared to their parental sdAbs (Table 3). The refolding percentage is slightly decreased in both EBOV-GP-G6-neg+ and EBOV-GP-H7-neg+ relative to their respective parental sdAbs. EBOV-GP-A8 and EBOV-GPA8-neg+ containing an unpaired Cys106 exhibited no refolding recovery after heat denaturation. The elimination of this Cys by substituting Ser in EBOV-GP-A8-fneg+ significantly increased the refolding capability from 0% up to 66% (Table 3).

**Table 3:** Comparison of Tm, refolding and binding kinetics for EBOV GP binders and their derivatives

Clone name	Tm (°C)	Refolding (%)	ka (1/Ms)	kd (1/s)	K_D_ (M)
EBOV-GP-G6	72	77	7.0 E+04	1.5E−04	2.0E−09
EBOV-GP-G6-neg+	87	67	3.5E+03	1.5E−04	4.4E−08
EBOV-GP-H7	70	97	1.7E+04	3.0E−04	1.7E−08
EBOV-GP-H7-neg+	87	85	3.5E+04	1.4E−03	4.0E−08
EBOV-GP-A8	57	0	3.1E+04	7.2E−05	2.3E−09
EBOV-GP-A8-neg+	73	0	6.1E+04	5.8E−05	9.5E−10
EBOV-GP-A8-fneg+	74	66	1.3E+05	9.1E−05	6.9E−10

Three of the GP binding sdAbs showing the highest melting temperatures and/or best affinities were subject to mutagenesis to improve their stability. Melting temperature and refolding ability were measured by CD

Binding affinity was measured using SPR and compared among parental sdAbs and mutants. Our results indicate that the binding affinity for EBOV-GP-G6-neg+ and -H7-neg+ is 2–5-fold worse than the respective parental sdAbs, while the EBOV-GP-A8-fneg+ has 5-fold better binding affinity than the parental A8 (Table 3). Disulfide bond addition has previously been found to negatively impact the binding affinity of some sdAbs as we observed for EBOV-GP-G6-neg+ and EBOV-GP-H7-neg+ [25], thus we were pleased that EBOV-GP-A8-neg+ and EBOV-GP-A8-fneg+ both possessed improved affinities relative to the parental EBOV-GP-A8. This difference would suggest that while we found these three sdAbs bind overlapping epitopes by SPR competition (Additional file 1: Figure S5), that their binding interactions with GP are not identical. We found no difference in binding affinity between GSKKK fusions and the neg+ mutants.

### Detection of EBOV GP and VLPs

The sdAbs were examined by both sandwich and direct binding assays to determine their ability to bind to EBOV GP and VLPs. Both bead-based MagPlex assays as well as ELISAs were utilized in these experiments. A lysine-containing tail termed GSKKK (GGGGSGGGGKKK) was fused to the C-terminus of the sdAbs aiming to either increase biotin incorporation for use as the detection molecule or to enhance directional immobilization onto the surface to improve the percentage of active antibody attached, with the goal of improving the detection limits for GP/VLP (Fig. 2d) [39].

**Fig. 4:**
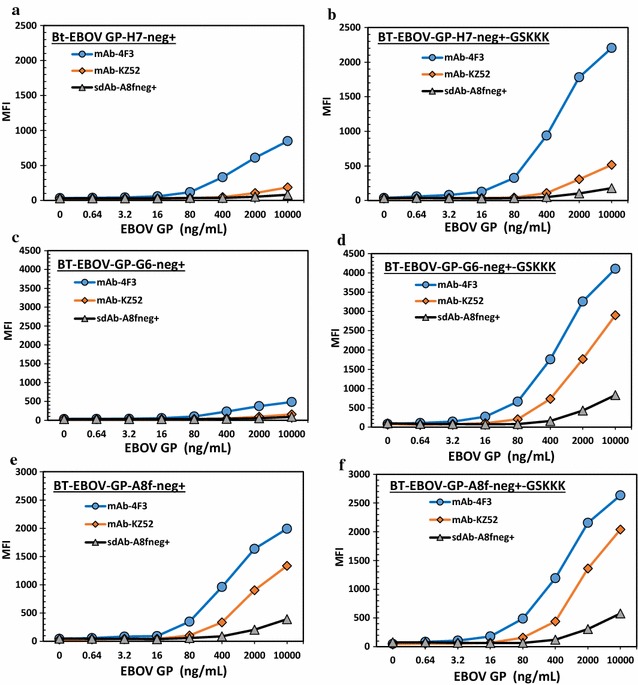
Comparison of EBOV GP binding for sdAb-GSKKK fusions. Bt-sdAb-neg+-GSKKK fusions (**b**, **d**, **f**) and Bt-sdAb-neg+ (**a**, **c**, **e**) were used as tracers to detect EBOV GP captured by conjugated mAb KZ52, mAb 4F3 and EBOV-GP-A8-fneg+

Multiplex MagPlex assays were used to detect GP captured by human EBOV monoclonal Ab (mAb), KZ52, mouse mAb 4F3 and EBOV GP sdAb mutant, EBOV-GP-A8-fneg+. Biotinylated sdAb (Bt-sdAb) mutants were then used as tracer Abs to detect the captured GP. In these experiments, mAb 4F3 was a better capture Ab than KZ52 (Fig. 4). Our results indicated adding the GSKKK tail to the sdAbs improved the detection limits from 5 to 25-fold. Thus, the GSKKK was effective in improving the sdAbs as tracer reagents (Fig. 4).

The addition of a positive tail was expected to facilitate the directional immobilization of sdAbs onto the negatively charged microsphere surface and thus improve the detection limits in that manner as well [39], unfortunately, we did not see the GSKKK fusion achieve this result. The cause of this is unclear, but perhaps the sdAb’s binding site on the GP is difficult for the surface immobilized sdAb to access efficiently.

**Fig. 5:**
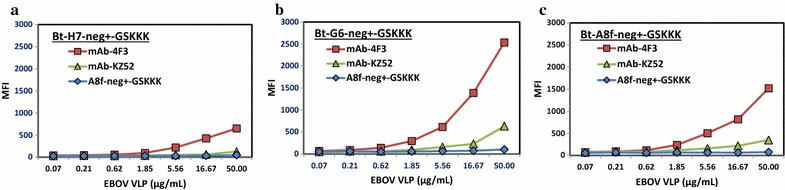
Binding of EBOV GP binders to EBOV VLPs. Three Bt-sdAb-neg+-GSKKK fusions were used as tracer to detect captured EBOV VLPs by conjugated mAb, KZ52, mAb 4F3, and EBOV-A8-fneg+-GSKKK. Among three fusions, Bt-G6-neg+-GSKKK **b** and Bt-A8-fneg+-GSKKK **c** have higher signals than Bt-H7-neg+-GSKKK **a** and both detect VLPs as low as 1.85 μg/mL

**Fig. 6:**
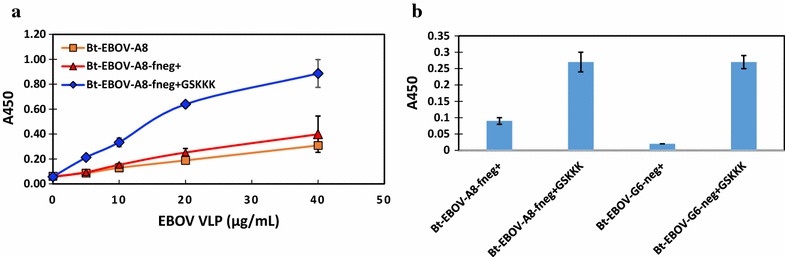
Comparison of VLP binding among EBOV-sdAbs and their corresponding engineered constructs using ELISA. **a** Binding of captured EBOV VLPs at various concentrations is assessed using Bt-EBOV-GP-A8 and its engineered constructs as tracers. **b** Comparison of binding of VLPs to EBOV-sdAbs and their GSKKK fusions

The binding of EBOV VLPs by the sdAbs and their fusions was also measured by MagPlex assay and ELISAs. Our results show that the Bt-sdAb-GSKKK fusions are able to bind to VLPs captured by mAb 4F3 and mAb KZ52. The sdAbs captured much less than mAbs, and EBOV-GP-A8-fneg+-GSKKK is shown in Fig. 5. As reporter reagents, both Bt-EBOV-GP-A8-fneg+-GSKKK and Bt-EBOV-GP-G6-neg+-GSKKK generate better binding signals than Bt-EBOV-GP-H7-neg+-GSKKK and detect captured VLPs as low as 1.85 μg/mL (Fig. 5). The binding of captured VLPs among EBOV-GP-A8, EBOV-GP-A8-fneg+, and EBOV-GP-A8-fneg+-GSKKK in increasing concentrations of VLPs confirms that the addition of GSKKK improves the detection significantly (Fig. 6a). Both Bt-EBOV-GP-A8-fneg+-GSKKK and Bt-EBOV-GP-G6-neg+-GSKKK fusions exhibit 2–10-fold better VLP binding signals than non-fusions (Fig. 6b). We have observed that the fusion of the GSKKK tail to the C-terminus of sdAbs does not alter physical properties or binding affinity. In this case, the addition of the GSKKK tail led to improved reporter reagents which generate better binding signals when used as tracer antibodies in both MagPlex assays and ELISAs (Figs. 4, 6). It is likely that ε-amines on the Lys residues of the tail recruit more biotins and result in more binding of streptavidin conjugate. While the biotinylation was performed on all the sdAbs identically, it will be necessary to quantify the degree of biotinylation to decipher whether the GSKKK tail is providing for a higher degree of biotinylation, which improves the effectiveness, or just provides a location that is well positioned for binding of the streptavidin conjugate by being well away from the binding site.

Construction of genetically linked sdAbs (diabodies) has proven a helpful strategy to increase the usefulness of sdAbs [40–42]. Although competition experiments indicate that EBOV-GP-G6, EBOV-GP-H7 and EBOV-GP-A8 bind to the same or overlapping epitopes (Additional file 1: Figure S5), GP is a multimer in solution and on the VLP surface. Incorporating the isolated anti-EBOV GP sdAbs into linked constructs to form homodimers or heterodimers may improve their performance as captures and tracers in sandwich assays and is a strategy that can be investigated in the future.

### Cross-reactivity analysis of sdAbs and mutants

The binding of GPs from denatured VLPs, including EBOV, SUDV, MARV, and purified recombinant GPs from EBOV and MARV by the GP sdAb mutants was assessed using western blotting analysis. EBOV-GP-A8-fneg+, EBOV-GP-G6-neg+ and EBOV-GP-H7-neg+ and their GSKKK fusions all bound to GP from denatured EBOV VLPs and recombinant EBOV GP, but not in SUDV VLPs, MARV VLPs or recombinant MARV GP (Additional file 1: Figure S6 and unpublished data).

Although cross-reactive mAbs against conserved GP epitopes among five Ebola virus species were developed [43], we do not see cross-reactivity of the selected EBOV GP binding sdAbs to SUDV or MARV VLPs using western blot analysis. It is likely that the selected EBOV GP sdAbs bind to an epitope only on EBOV GP (Additional file 1: Figure S6). Polyclonal antibodies against EBOV VLPs show that GPs from denatured VLPs appear as multiple bands due to the extent of glycosylation with sizes that are between 62 and 110 kDa. The recombinant GP exhibits a similar profile except that fewer bands were obtained, with sizes between 60 and 70 kDa on western blots using commercial mouse anti-GP antibody. Our results suggest that the selected GP binders bind preferentially to less glycosylated GP bands with the sizes of 60 and 70 kDa from VLPs, however they also bind the heavily glycosylated GPs with the size more than 100 kDa in purified recombinant GP (Additional file 1: Figure S6).

## Conclusion

We prepared an immune phage display library from llamas immunized with killed EBOV and recombinantly produced EBOV GP, and successfully selected sdAbs that bind recombinant EBOV GP with ~ nM binding affinity. These sdAbs also bind to EBOV VLPs specifically. We incorporated changes including a non-canonical disulfide bond and negative charges in FR1 which resulted in Tm increases of at least 15 °C. The elimination of the unpaired non-canonical Cys106 in EBOV-GP-A8-fneg+ dramatically restored its refolding ability, going from 0 to 66%. In addition, the fusion of a C-terminal GSKKK tail increased the detection signals for EBOV VLP/GP approximately 2–25-fold relative to the parental clone when serving as a tracer antibody. We postulate that EBOV-GP-A8-fneg+-GSKKK with a sub-nM affinity to GP, 74 °C Tm and the best VLP binding ability is a prime candidate for further evaluation to determine its utility in diagnostic or therapeutic applications for EBOV.

## Methods

### Antibodies, antigens and reagents

The mouse mAb 4F3 and human mAb KZ52 both recognize EBOV GP and were from IBT Bioservices (Gaithersburg, MD). Recombinant GP without transmembrane domain (from EBOV and Marburg virus) as well as the EBOV, SUDV, and Marburg VLPs used in the western blotting experiment were also from IBT Bioservices. Killed EBOV was acquired from the (now defunct) Critical Reagents Program. In addition, the following reagent was obtained through BEI Resources, NIAID, NIH: Zaire Ebola virus Mayinga, Gamma-Irradiated, NR-31807.

Most reagents were purchased from Sigma-Aldrich (St Louis, MO), VWR, or Thermo Fisher Scientific. Enzymes and cloning reagents were purchased from New England Biolab (Ipswich, MA) unless specified.

### Immunization and construction of phage display library

A llama was immunized 5 times, 14 days apart, with 100 μg killed EBOV from the Department of Defense Critical Reagents Program followed 21 days later by one immunization of killed EBOV from BEI Resources and finally 14 days later boosting the animal twice (14 days apart) with recombinant EBOV GP. Peripheral blood lymphocytes in the buffy coat were isolated using a Ficoll separation method and the total RNA was isolated using QIAamp RNA Blood Mini kit (Qiagen Inc, Valencia, CA) according to manufacturer’s protocol. cDNA was obtained by reverse transcribing the total RNA using superscript RTIII and oligo-dT (both from Life Technologies, Grand Island, NY) according to manufacturer’s protocol. The heavy chain variable domain fragments were amplified from PCR reaction using degenerated primers [44] and our previously published modified protocol [7].

The immune library derived from a llama immunized with killed EBOV and recombinant GP was obtained by cloning the amplified variable domain fragments into the phage display vector, pECAN21 [7, 8]. Gel purified variable domain PCR fragments and pECAN21 DNA cut with SfiI (New England Biolab Inc, Ipswich, MA) were ligated overnight at 15 °C with a 3:1 ratio of insert to vector. The ligation mixture was then transformed to XL1 Blue cells (Agilent Technologies Inc, Clara, CA) using electroporation. Phage displaying sdAbs were prepared from the library according to the previous protocol [45]. Representative clones were sequenced to assess the library quality and diversity. Library sequencing was performed by Eurofins Genomics and analysis of the library sequences was as previously described [46].

### Biopanning, selection and sequence analysis of EBOV GP potential binders

Panning was carried out using a procedure similar to previous work [7, 45]. The recombinant GP at the concentration of 10 µg/mL was coated onto 4 wells of a 96-well plate at 4 °C overnight. The coated wells were then washed with PBS supplemented with 0.05% Tween 20 (PBST) and incubated with phage displaying sdAbs amplified from immunized phage display library. Following extensively washing wells with PBST and PBS, the binding phages were then eluted by 100 mM triethylamine, which was subsequently neutralized by adding 1 M Tri–HCl, pH 8.0. The eluted phage titer was determined by infecting the log phase XL1 Blue and referred to as R1 eluted phage. The eluted phage were also grown and replicated in XL1 blue to obtain higher titer of amplified phage, which was referred as R1 amplified phage. R1 amplified phage were then used to repeat the above panning procedure once to obtain R2 eluted phage and R2 amplified phage, and twice to obtain R3 eluted phage and R3 amplified phage. Bacterial colonies were obtained by infecting XL1 blue with R2 and R3 eluted phage and individual colonies were inoculated into wells in a 96-well plate. Approximately 198 colonies were grown to conduct monoclonal phage ELISA. Binding was assessed using the target GP as well as an irrelevant antigen (BSA). Colonies giving a higher absorbance at 450 nm on the target versus irrelevant antigen were selected and grown for plasmid purification. Plasmid DNA was sent out for sequencing (Eurofins Genomics, Louisville, KY). Amino acid sequences were obtained and aligned using multalin (http://multalin.toulouse.inra.fr/multalin/) to compare the variations in the complementarity determine regions (CDRs) [47].

### Expression and purification of potential binders

Representative sdAb genes from each sequence family were subcloned into a periplasmic expression vector, pET22b, for protein preparation and transformed into Turner (DE3) *E. coli* strain (EMD Millipore, Billerica, MA). The expression and purification procedure was performed according to the previously published protocol [48, 49]. Following IMAC extraction, sdAbs were further purified from other protein contaminates or aggregates through gel filtration chromatography using a Superdex 75 10/300 GL column (GE Healthcare) on a BioLogic DuoFLow chromatography system (Bio-Rad). Monomeric sdAb concentration was determined using BCA protein kit (Pierce) or a NanoDrop 1000 instrument.

### Modification of GP binders

DNA fragments encoding modified amino acid sequences were synthesized by Eurofins Genomics and later subcloned into pET22b expression vector for further expression and purification. To construct sdAb-GSKKK fusion, NcoI-NotI SdAb fragments were ligated into GSKKK-pET22b plasmids cut with NcoI and NotI. The sequences were verified by Sanger sequencing and transformed into Tuner (DE3). Sequence modified proteins and fusions were subsequently purified according to the above section.

### Measurement of Tm by circular dichroism (CD) and fluorescent dye melt assay

Protein samples were diluted to 22 µg/mL in deionized water and placed in a quartz cuvette with 1 cm path length. CD was measured at an ultraviolet wavelength between 200 and 210 nm using a Jasco J-815 Spectropolarimeter. Samples were heated from 25 to 95 °C at a rate of 2.5 °C/min. A cooling stage at the same rate was used to assay the amount of refolding after denaturation. The Tm is taken to be the inflection point of the S-shaped denaturation curve. The refolding ability is calculated as the change in CD magnitude upon cooling divided by the change in magnitude upon heating, expressed as a percentage.

The Fluorescent dye-based melting assay was performed as described previously [45]. Each of the sdAbs was diluted to a concentration of 500 µg/mL in a final volume of 20 µL PBS and Sypro Orange dye was added a dilution of 1:1000. Samples were measured in triplicate using a StepOne Real-Time PCR machine (Applied Biosystems, Foster City, CA). The heating program was run in continuous mode from 25 to 99 °C at a heating rate of 1% (~ 2 °C/min), and data was recorded using the ROX filter. The melting point was determined to be the peak of the first derivative of the fluorescence intensity. Each measurement was performed in triplicate, all three replicates giving essentially identical values for the melting temperature.

### Surface plasmon resonance (SPR)

Surface plasmon resonance affinity and kinetics measurements were performed using the ProteOn XPR36 (Bio-Rad). Lanes of a general layer compact (GLC) chip were individually coated with GP or left uncoated initially and then coated with GP for additional tests. Immobilization of the proteins was performed using dilution to 20 µg/mL in 10 mM acetate buffer pH 5.0 and attached to the chip following the standard 1-ethyl-3-(3-dimethylaminopropyl)carbodiimide hydrochloride (EDC)/*N*-hydroxysulfosuccinimide (sulfo-NHS) coupling chemistry available from the manufacturer. Binding kinetics of each sdAb was tested at 25 °C by flowing six concentrations typically varying from 3000 to 0 nM at 100 μL/min for 90 s over the antigen coated chip and then monitoring dissociation for 600 s. Following each run, the chip was regenerated by flowing 0.085% phosphoric acid (~ pH 3.0) across the surface for 18 s. Data analysis was performed with ProteOn Manager 2.1 software, corrected by subtraction of the zero antibody concentration column as well as interspot correction. The standard error on the fits was less than 10%. Binding constants were determined using the Langmuir model built into the analysis software. The competition experiments were performed by flowing EBOV-GP-A8 over three of the sensor chip’s six lanes to saturate those spots. Then three other sdAbs were flowed over two lanes each, one previous saturated with EBOV-GP-A8 and one unreacted. The amount of signal differential resulting is indicative of the amount of binding inhibition, see Additional file 1: Figure S5.

### MagPlex sandwich immunoassays

MagPlex assays were performed essentially as described previously [23]. Briefly, MagPlex beads were coated with the desired monoclonal Abs or sdAbs using the recommended two step EDC/sulfo-NHS chemistry. The biotin-labeled sdAbs (Bt-sdAbs) were prepared by using a 10-fold molar excess of NHS-LC-LC-biotin, after 30 min the excess biotin was removed using a Zeba spin 7 K desalting column (ThermoFisher). The protein-coated MagPlex beads (~ 100/set) were mixed with various concentrations of GP or EBOV VLPs diluted into PBSTB (PBS + 0.05% Tween (PBST) and 1 mg/mL bovine serum albumin (BSA)) in the wells of a 96-well polystyrene round bottom microtiter plate. After 30 min the beads were washed by placing the plate on a 96f magnet (BioTek, Winooski, VT) and washing three times with PBST. The beads were then incubated with the 1 µg/mL Bt-sdAb as indicated. After 30 min the beads were washed 3 times and then, to complete the fluorescent sandwich assay, the beads were incubated for 30 min with 2.5 µg/mL streptavidin conjugated phycoerythrin (SAPE, Columbia Biosciences, Frederick, MD). After a final wash, the binding was measured on the MAGPIX instrument (Luminex Corp., Austin, TX). The median value obtained by the evaluation of ≥ 50 microspheres for each set plotted, and error bars plotted as the standard error of the mean (SEM), which is typically less than ± 10% the mean.

### Enzyme-linked immunosorbent assay (ELISA*)*

Mouse monoclonal Ab, 4F3 was coated onto wells in a 96-well plate at the concentration of 2 µg/mL at 4 °C overnight. Next day, wells were washed with 3× PBST and blocked with PBST supplemented with 2% milk powder (2% MPBST) at room temperature (RT) for 1 h, followed by the addition of EBOV VLP at various concentrations of 2, 5, 10, 20, and 40 µg/mL at RT for 1 h. Bt-sdAbs were then subsequently added onto wells at the concentration of 2 µg/mL at RT for 1 h after washing wells with PBST and PBS. Horseradish peroxidase (HRP) conjugated Streptavidin was then added into wells at the concentration of 1 µg/mL at RT for 1 h. Peroxidase substrate, SureBlue TMB-1 component (KPL, Gaithersburg, MD) was added to each well and the reaction was stopped by the addition of 1 M HCl according to the manufacturer’s protocol before measuring the absorbance at 450 nm.

### Western blotting

Approximately 5 µg of EBOV VLP containing GP, NP and VP40 (IBT Bioservices, Gaithersburg, MD) and 5 μg of EBOV rGPdTM in 15 µL of 1× LDS sample buffer supplemented with 1× reducing agent was loaded onto pre-casted 4–12% BIS–TRIS NuPAGE gel in 1× MES-SDS running buffer, followed by applying electric current through the gel to separate the proteins by size. SeeBlue plus 2 prestained standard was loaded in separate lane as a size marker. Following the manufacturer’s protocol, separated proteins on the gel were then transferred to a PVDF membrane on ice. The blot was then washed with 1× TBST (10 mM Tris pH 7.5, 150 mM NaCl and 0.01% Tween 20), and was subsequently blocked with 5% milk in 1× TBST (TBSTM) at room temperature (RT) for 1 h. Approximately 1–5 µg/mL of primary antibody was then added to the blot at RT for 1 h before washing with TBSTM for 15 min three times. Horseradish peroxidase conjugated secondary antibody at a concentration of 1–2 µg/mL was then added onto the blot at RT for 1 h, followed by washing with TBST for 15 min three times. The luminescent substrate was then applied to the blot and incubated for 5 min and the images were captured by using a GEL doc XR + system.

## Additional file

**Additional file 1: Figure S1.** Biopanning against EBOV GP. The titer of eluted phage was determined at the end of each round of biopanning. **Figure S2.** Thirty-four potential GP-binding sdAb sequences selected from the second and third rounds of panning against EBOV GP. Sequences were divided into families based on similarity of CDR sequences. **Figure S3.** Representative measurements of binding kinetics and K_D_ using SPR biosensor. **Figure S4.** Table of IMGT number and corresponding amino acid at each position for clones EBOV-GP-A8, EBOV-GP–H7 and EBOV-GP-G6. **Figure S5.** Competition data assessing the ability of the sdAbs to bind GP that was pre-bound with EBOV-GP-A8. **Figure S6.** Western blotting analysis of GP and VLP binding specificity and cross reactivity for EBOV-GP-G6-neg+-GSKKK.
