# Supplementary material for: Selection, characterization, and thermal stabilization of llama single domain antibodies towards Ebola virus glycoprotein
Source: Microb Cell Fact. 2017 Dec 12;16:223. doi: 10.1186/s12934-017-0837-z (PMC5726015; doi:10.1186/s12934-017-0837-z)
Supplement: Supplementary file 1 — Additional file 1: Figure S1. Biopanning against EBOV GP. The titer of eluted phage was determined at the end of each round of biopanning. Figure S2. Thirty-four potential GP-binding sdAb sequences selected from the second and third rounds of panning against EBOV GP. Sequences were divided into families based on similarity of CDR sequences. Figure S3. Representative measurements of binding kinetics and KD using SPR biosensor. Figure S4. Table of IMGT number and corresponding amino acid at each position for clones EBOV-GP-A8, EBOV-GP–H7 and EBOV-GP-G6. Figure S5. Competition data assessing the ability of the sdAbs to bind GP that was pre-bound with EBOV-GP-A8. Figure S6. Western blotting analysis of GP and VLP binding specificity and cross reactivity for EBOV-GP-G6-neg+-GSKKK. [file 12934_2017_837_MOESM1_ESM.pdf]

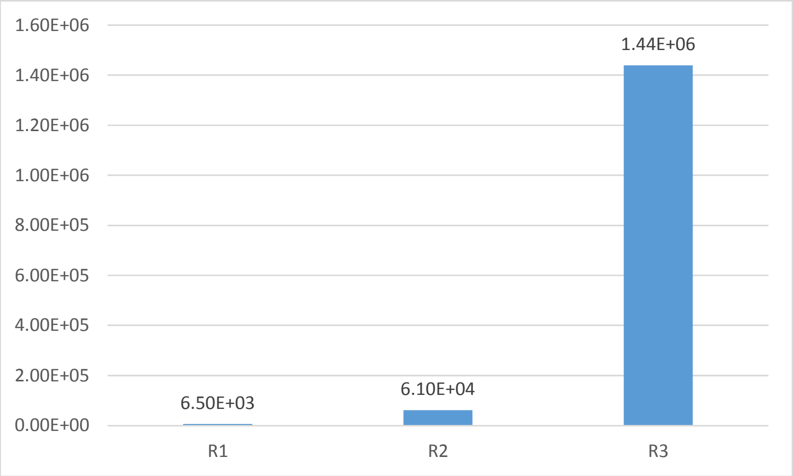

Fig S1. Biopanning against EBOV GP. The titer of eluted phage was determined at end of each round of biopanning. Each panning round used the same number of inputted phage (~$5x10^{11}$).

```
I    R2A1   EVQLQASGGGLVQARGSLQLSCAAPGRPVRTYTMGWFRQAPGKEREFVAANNWRG--TRYAGSVRGRFSISRDNSKNT--VYLQMNSLKPEDTAVYYCACAVTADLLLSGNYFRGDDYDYWGQGTQVTVS
     R3B10  EVQLQASGGGLVQARGSLQLSCAAPGRPVRTYTMGWFRQAPGKEREFVAANNWRG--TRYAGSVRGRFSISRDNSKNT--VYLQMNSLKPEDTAVYYCACAVTADLLLSGNYFRGDDYDYWGQGTQVTVS
     R2E4   EVQLQASGGGLVQAGGSLQLSCAAPGRPVRTYTMGWFRQAPGKEREFVAANNWRG--TRYAGSVRGRFSISRDNSKNT--VYLQMNSLKPEDTAVYYCACAVTADLLLSGNYFRGDDYDYWGQGTQVTVS
     R2G5   EVQLQASGGGLVQAGGSLQLSCAAPGRPVRTYTMGWFRQAPGKEREFVAANNWRG--TRYAGSVRGRFSISRDNSKNT--VYLQMNSLKPEDTAVYYCACAVTADLLLSGNYFRGDDYDYWGQGTQVTVS
     R2G10  EVQLQASGGGLVQAGGSLQLSCAAPGRPVRTYTMGWFRQAPGKEREFVAANNWRG--TRYAGSVRGRFSISRDNSKNT--VYLQMNSLKPEDTAVYYCACAVTADLLLSGNYFRGDDYDYWGQGTQVTVS
     R3D12  EVQLQASGGGLVQAGGSLQLSCAAPGRPVRTYTMGWFRQAPGKEREFVAANNWRG--TRYAGSVRGRFSISRDNSKNT--VYLQMNSLKPEDTAVYYCACAVTADLLLSGNYFRGDDYDYWGQGTQVTVS
     R3A8   EVQLQASGGGLAQAGGSLQLSCAAPGRPVRTYTMGWFRQAPGKEREFVAANNWRG--TRYAGSVRGRFSISRDNSKNT--VYLQMNSLKPEDTAVYYCACAVTADLLLSGNYFRGDDYDYWGQGTQVTVS
     R3H8   EVQLQASGGGLVQAGGSLQLSCAAPGHPVRTYTMGWFRQAPGKEREFVAANNWRG--TRYAGSVRGRFSISRDNSKNT--VYLQMNSLKPEDTAVYYCACAVTADLLLSGNYFRGDDYDYWGQGTQVTVS
     R2B4   EVQLQASGGGLVQAGGSLQLSCAAPGRPVRTYTMGWFRQAPGKEREFVAANNWRG--TRYAGSVRGRFSISRDNSKNT--VYLQMNSLKPEDTAVYYCACAETADLLLSGNYFRGDDYDYWGQGTQVTVS
     R3E9   EVQLQASGGGLVQAGGSLQLSCAAPGRPVRTYTMGWFRQAPGKEREFVAANNWRG--TRYAGSVRGRFSISRDNSKNT--VYLQMNSLKPEDTAVYYCACAETADLLLSGNYFRGDDXDYWGQGTQVTVS
     R3G10  EVQLQASGGGSVQAGGSLQLSCAAPGRPVRTYTMGWFRQAPGKEREFVAANNWRG--TRYAGSVRGRFSTSRDNSKNT--VYLQMNSLKPEDTAVYYCACAVTADLLLSGNYFRGDDYDYWGQGTQVTVS
     R3C11  DVQLQASGGGLVQAGGSLQLSCAAPGRPVRTYTMGWFRQAPGEEREFVAANNWRG--TRYAGSVRGRFSVSRDNSKNT--VYLQMNSLKPEDTAVYYCACAVTADLLLSGNYYRGDDYDYWGLGTQVTVS
     R2B6   DVQLQASGGGLVQAGGSLQLSCAAPGRPVRTYTMGWFRQAPGMEREFVAANNWRG--TRHAGSVRGRFSISRDNSKNT--VYLQMNSLKPEDTAVYYCACAVTADLLLSGNYFRGDDYDYXGQGTQVTVS
II   R2E6   DVQLQASGGGLVQAGGSLQLSCAAPGRPVRTYTMGWFRQAPGMEREFVAANNWRG--TRHAGSVRGRFSISRDNSKNT--VYLQMNSLKPEDTAVYYCACAVTADLLLSGNYFRGDDYDYWGQGTQVTVS
     R2B10  EVQLQASGGGLVQAGGSLRLSCTASLRAFSTYTMGWFRQAPGKEREFVAASNWRG--TDFHDSVKGRFIISRDNTKKT--VDLQMNSLKPEDTAIYYCAAD-------GSTWLKRSDYSYWGQGTQVTVS
     R2B5   EVQLQASGGGLVQAGGSLRLSCTASLRAFSTYTMGWFRQAPGKEREFVAASNWRG--TDFHDSVKGRFIISRDNTKKT--VDLQMNSLKPEDTAIYYCAAD-------GSTWLKRSDYSYWGQGTQVTVS
     R2E12  DVQLQASGGGLVQAGGSLRLSCTASLRAFSTYTMGWFRQAPGKEREFVAASNWRG--TDFHDSVKGRFIISRDNTKKT--VDLQMNSLKPEDTAIYYCAAD-------GSTWLKRSDYSYWGQGTQVTVS
     R2D1   EVQLQASGGGSVPPGGSLRLSCTASLRAFSTYTMGWFRQAPGKEREFVAASNWRG--TDFHDSVRGRFIISRDNTEKT--VDLQMNSLKPEDTAIYYCAAD-------GSTWLKRSDYSYWGQGTQVTVS
III  R3D10  EVQLQASGGGSVPPGGSLRLSCTASLRAFSTYTMGWFRQAPGKEREFVAASNWRG--TDFHDSVRGRFIISRDNTEKT--VDLQMNSLKPEDTAIYYCAAD-------GSTWLKRSDYSYWGQGTQVTVS
     R2B11  EVQLQASGGGLVQTGGSLRLACAGSGRSFNNYGMGWFRQAPGKERQFVAAISGNDHTTHYSDSVKGRFTISRDNAKTT--VYLQMNSLKPEDTAVYYCAADRLGSF--FFPRASPSDYGYWGQGTQVTVS
     R3B7   DVQLQASGGGLVQTGGSLRLACAGSGRSFNNYGMGWFRQAPGKERQFVAAISGNDHTTHYSDSVKGRFTISRDNAKST--VYLQMNSLKPEDTAVYYCAADRLGSF--FFPRASPSDYGYWGQGTQVTVS
     R2G6   DVQLQASGGGLVQAGGSLRISCAASGGALSDYNMGWFRQAPGKEREFVAVVTWSGGSTRYADSVNGRFTISRDNAKNT--VYLQMNSLKPEDTAVYYCAAAPLTPF--Q-SLGDMKAYNYWGQGTQVTVS
     R3H7   DVQLQASGGGLVQAGGSLRLSCAASSGTFSSYTMGWFRQAPGKERELVARLSQS-LTTLYADSVKGRFTISRDNAENT--VYLQMNSLKPEDTGVYYCAAHPGTYG--SAWLISPHDYAYWGQGTQVTVS
IV   R3F12  DVQLQASGGGLVQAGGSLRLSCAASSGTFSSYTMGWFRQAPGKERELVARLSQS-LTTLYADSVKGRFTISRDNAENT--VYLQMNSLKPEDTGVYYCAAHPGTYG--SAWLISPHDYAYWGQGAQVTVS
     R2C12  EVQLQASGGGLVQPGGSLRLSCTASGSIFSINAMGWYRQTPRKERELVAAISEGG-STKYGDSVKGRFTISRDFAKNTQMVYLQMNSLKAEDTAVYYCKAQTEG----PIADWGTSQYEYWGQGTQVTVS
     R2H1   EVLLQASGGGLVQPGGSLRLSCAASGLIFSINVMGWYRQTPGKQRELVASISRDD-STKYADSVKGRFTISRDNAKNT--VYLQMNSLKPEDTAVYYCNTQTEG----PVADWGTSHYDYWGQGTQVTVS
     R2E7   EVQLQASGGGLVQPRGSLRLSCAASGSIFSINAMGWYRQTPEKQRELVATISSSG-STKYGDSVKGRFTISRDNAKNT--VYLQMNSLKPEDTAVYYCNTQTAG----LVADWGTSHYDYWGQGVQVTVS
     R2H11  EVQLQASGGGLVQAGGSTRLSCAGSGSIFSINAMGWYRQTPEKQRELVATISSSG-STKYGDSVKGRFTISRDNAKNT--VYLQMNSLKPEDTAVYYCNTQTAG----PVADWGTSHYDYWGQGVQVTVS
V    R2D10  EVQLQASGGGLVEPGGPLRLSCAVSGINFNINHWGWYRQAPGKQREWVATIAIGG-ATDYADSLKGRFTISRDNMKNT--VYLQMNSLRPEDTAVYYCN----G----VGSKWS----DRWGQGTQVTVS
     R2G2   EVQLQASGGGLVEPGGSLRLSCAVSGINFNINHWGWYRQAPGKQREWVATIAIGG-ATDYADSLKGRFTISRDNMKNT--VYLQMNSLRPEDTAVYYCN----G----VGSKWS----DRWGQGTQVTVS
     R3F8   EVQLQASGGGLVEPGGSLRLSCAVSGINFNINHWGWYRQAPGKQREWVATIAIGG-ATDYADSVKGRFTISRDNMKNT--VYLQMNSLRPEDTAVYYCN----G----VGSKWS----DRWGQGTQVTVS
     R2G11  EVQLQASGGGLVEPGGSLRLSCAVSGINFNINHWGWYRQAPGKQREWVATIAIGG-ATDYADSLKGRFTISRDNMKNT--VYLQMSSLRPEDTAVFYCN----G----VGSKWS----DRWGQGTQVTVS
     R2G3   EVQLQASGGGLVEPGGSLRLSCAVSGINFNINHWGWYRQAPGKQREWVATIAIGG-ATDYADSLKGRFTISRDNMKNT--VYLQMNSLRPEDTAVYYCN----G----VGSKWS----VRWGQGTQVTVS
     R2C8   EVQLQASGGGLVEPGGSLRLSCAVSGINFNINHWGWYRQAPGKQREWVATIAIGS-ATDYADSLKGRFAISRDNMKNT--VYLQMNSLRPEDTAVYYCN----G----VGSKWS----DRWDQGTQVTVS
Consensus   #VQLQASGGGLV#.GGSLrLSCaasg..fs.y.mGW%RQaPgK#RE.VA.i...g..T.%adSvkGRFtISRDN.knT  VyLQMNSLkPEDTA!YYCa....g.............y.yWGQGtQVTVS
```

FigS2. Thirty four potential GP sdAb sequences selected from the second and third rounds of panning against EBOV GP. R2 signifies sequences selected in round 2 while R3 sequences were isolated from round 3.

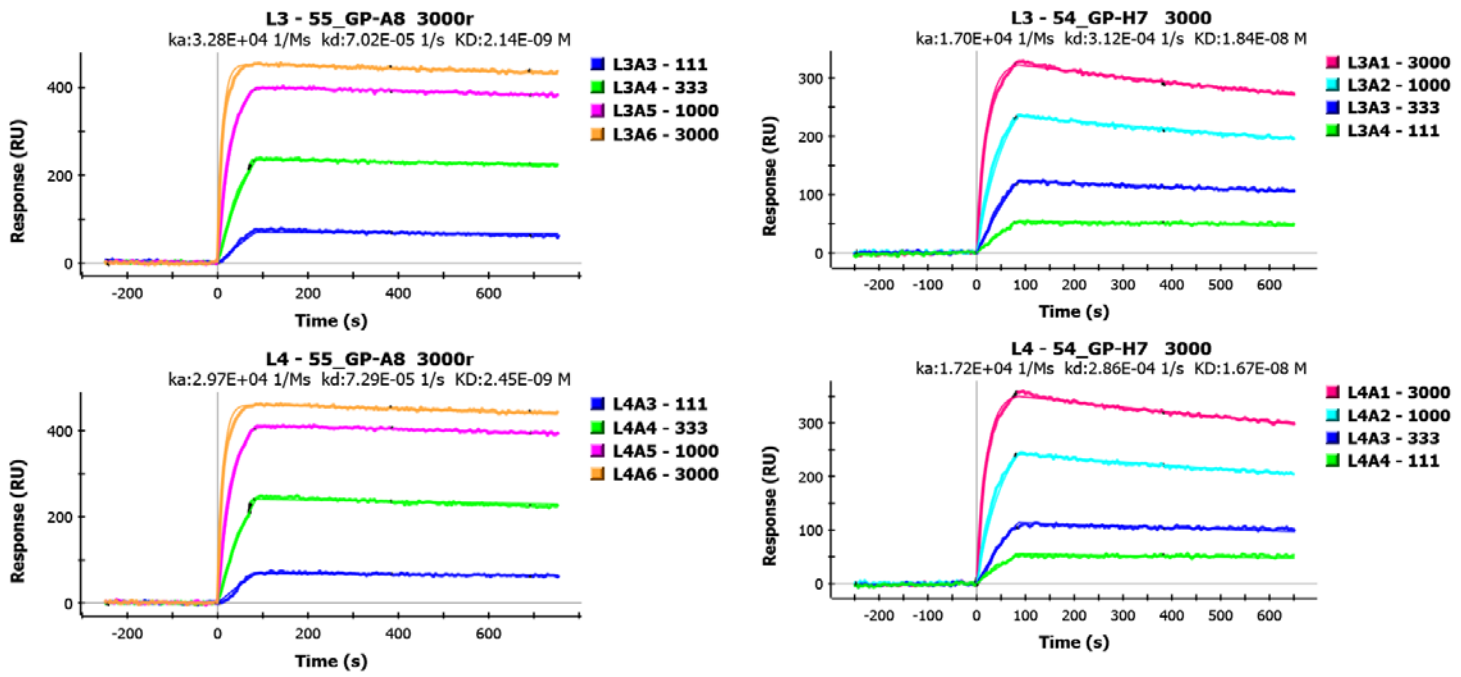

Fig. S3. Measurements of KD using SPR biosensor. The surface was coated with EBOV GP first and a serial dilution of purified sdAbs (3000, 1000, 333, and 111 nM) were then injected. Duplicate data is shown above for EBOV-GP-A8 on the left and EBOV-GP-H7 on the right.

IMGT number	EBOV-GP-H7	EBOV-GP-G6	EBOV-GP-A8
1	D	D	E
2	V	V	V
3	Q	Q	Q
4	L	L	L
5	Q	Q	Q
6	A	A	A
7	S	S	S
8	G	G	G
9	G	G	G
10	-	-	-
11	G	G	G
12	L	L	L
13	V	V	A
14	Q	Q	Q
15	A	A	A
16	G	G	G
17	G	G	G
18	S	S	S
19	L	L	G
20	R	R	Q
21	L	I	L
22	S	S	S
23	C	C	C
24	A	A	A
25	A	A	A
26	S	S	P
27	S	G	G
28	G	G	R
29	T	A	P
30	F	L	V
31	-	-	-
32	-	-	-
33	-	-	-
34	-	-	-
35	S	S	R
36	S	D	T
37	Y	Y	Y
38	T	N	T
39	M	M	M
40	G	G	G
41	W	W	W
42	F	F	F
43	R	R	R
44	Q	Q	Q
45	A	A	A
46	P	P	P
47	G	G	G
48	K	K	K
49	E	E	E
50	R	R	R
51	E	E	E
52	L	F	F
53	V	V	V

54	A	A	A
55	R	V	A
56	L	V	N
57	S	T	N
58	Q	W	W
59	S	S	-
60	-	-	-
61	-	-	-
62	-	G	-
63	L	G	R
64	T	S	G
65	T	T	T
66	L	R	R
67	Y	Y	Y
68	A	A	A
69	D	D	G
70	S	S	S
71	V	V	V
72	K	N	R
73	-	-	-
74	G	G	G
75	R	R	R
76	F	F	F
77	T	T	S
78	I	I	I
79	S	S	S
80	R	R	R
81	D	D	D
82	N	N	N
83	A	A	S
84	E	K	K
85	N	N	N
86	T	T	T
87	V	V	V
88	Y	Y	Y
89	L	L	L
90	Q	Q	Q
91	M	M	M
92	N	N	N
93	S	S	S
94	L	L	L
95	K	K	K
96	P	P	P
97	E	E	E
98	D	D	D
99	T	T	T
100	G	A	A
101	V	V	V
102	Y	Y	Y
103	Y	Y	Y
104	C	C	C
105	A	A	A
106	A	A	C
107	H	A	A

108	P	P	V
109	G	L	T
110	T	T	A
111	Y	P	D
111A	G	F	L
111B	S	Q	L
111C	A	S	L
112D	W	-	S
112E	-	-	G
112D			N
112C	L	L	Y
112B	I	G	F
112A	S	D	R
112	P	M	G
113	H	K	D
114	D	A	D
115	Y	Y	Y
116	A	N	D
117	Y	Y	Y
118	W	W	W
119	G	G	G
120	Q	Q	Q
121	G	G	G
122	T	T	T
123	Q	Q	Q
124	V	V	V
125	T	T	T
126	V	V	V
127	S	S	S
128	S	S	S

Fig. S4. Table of IMGT number and corresponding amino acid at each position for clones EBOV-GP-A8, EBOV-GP-H7 and EBOV-GP-G6. CDR regions are highlighted in red for CDR1, blue for CDR2, and green for CDR3.

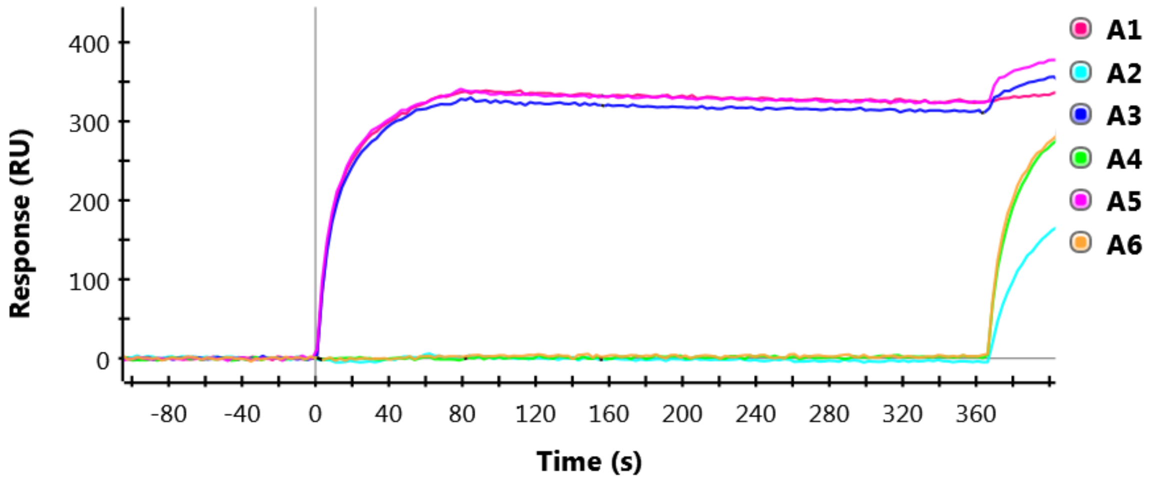

SdAb	% Inhib. vs A8
EBOV-GP-H7	68
EBOV-GP-D1	82
EBOV-GP-B5	80
EBOV-GP-B11	87
EBOV-GP-G6	97
EBOV-GP-E7	0
EBOV-GP-C12	0

Fig S5. Binding competition determination. First an SPR chip was coated with EBOV GP. Next EBOV-GP-A8 was flowed over three lanes (lanes 1, 3, and 5); buffer only was flowed over lanes 2, 4, and 6. Finally various anti-EBOV GP sdAbs were flowed over pairs of lanes: one lane blocked by pre-binding of EBOV-GP-A8 and one unblocked (buffer only in the previous step). To determine inhibition we compared sdAb binding to the two surfaces. The graph above shows data for EBOV-GP-H7 (red and light blue), EBOV-GP-D1 (dark blue and green), and EBOV-GP-B5 (pink and orange). The adjoining table shows the calculated percent inhibition versus A8 for the 3 sdAbs shown on the graph above and sdAbs determined by performing additional tests.

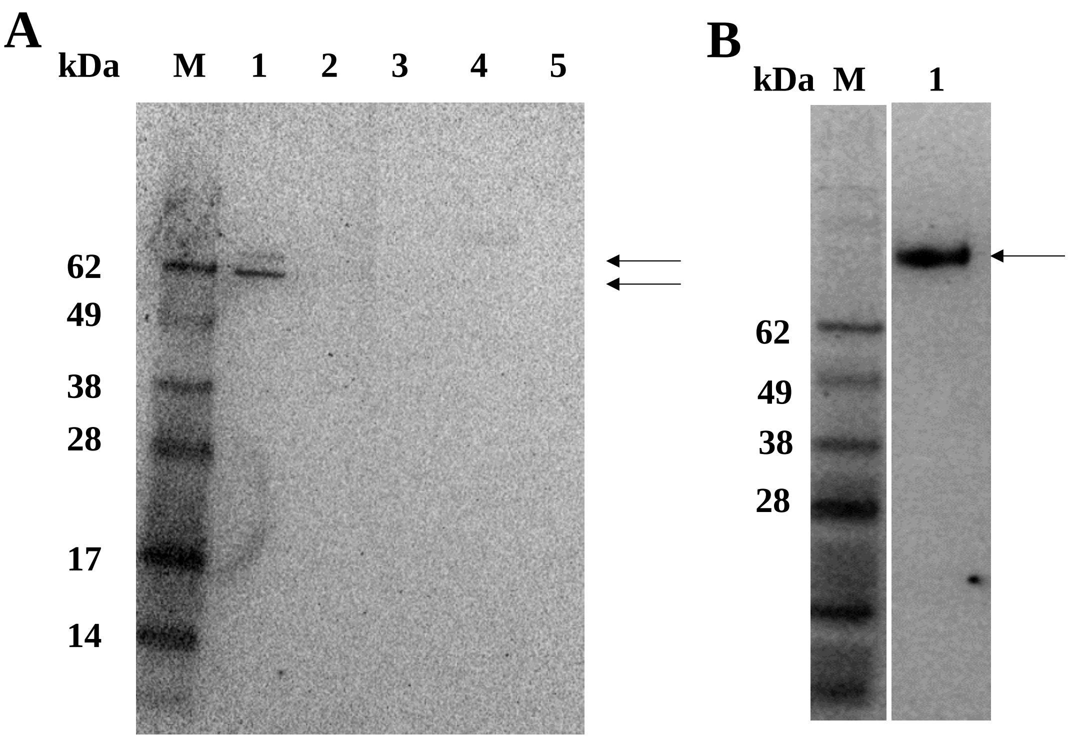

Fig. S6. Western blotting analysis of GP and VLP binding specificity and cross reactivity for G6-neg+-GS3K. A. Lane 1 represents EBOV VLPs, where as GP indicated by arrows. Lanes 2 and 3 represent the SUDV VLPs and MARV VLP respectively. Lanes 4 and 5 represent EBOV GP and MARV GP, respectively. Lane M is the size marker. B. The binding of A8-fneg+-GS3K to EBOV GP. Lane 1 represents EBOV GP as indicated by the arrow.
